# Inoculations against Swine-erysipelas (Rothlauf)

**Published:** 1897-08

**Authors:** 


### ABSTRACTS FROM FOREIGN JOURNALS.

### GERMAN.

UNDER THE DIRECTION OF J. PRESTON HOSKINS,
PRINCETON, N. J.

INOCULATIONS AGAINST SWINE-ERYSIPELAS (ROTHLAUF).—The tables of statistics for the quarter-year, July–September, 1896, contain numerous notices in regard to preventive inoculation against swine-erysipelas which certainly deserve to become more widely known, because they give valuable contributions toward judging the worth of these inoculations. The deaths mentioned in the following lists were caused by erysipelas.

**You can visit famous homes of noted thoroughbreds. Go and see St. Blaise, at Nashville.**

In the Königsberg district inoculation with Pasteur's lymph:

1. 36 swine inoculated. Five or six days later 17 died.

2. 22 swine inoculated. After two months 1 death.

3. 85 swine inoculated. One death after the first inoculation. Eight weeks after the second inoculation 22 died.

4. 60 swine inoculated. Many of the same became stiff and crippled in the hind legs after inoculation. A certain number, not specified, died with all the symptoms of erysipelas.

5. 30 swine inoculated. Sixteen died three weeks after the second inoculation.

6. 8 swine died after the first inoculation, 12 after the second, besides 6 inoculated swine which had been purchased elsewhere.

7. All 50 inoculated swine remained healthy. One hog which had been bought died on the day after purchase.

8. All the swine on the farm inoculated. Three months later 14 hogs sick with erysipelas had to be killed.

In the district of Gumbrunen: On one farm 13 hogs which had been inoculated with Pasteur's lymph in April died between July and September. In the Dunzig district, in two places, no preventive effect could be noticed after inoculation with Pasteur's lymph. In several places in the Dunzig district herds of swine were inoculated according to the process of Lorenz. No record occurs of an outbreak of erysipelas in these herds. In the district of Bromberg: In a herd of swine inoculated with porkosan, 10 died. In the district of Tundeburg: 54 hogs inoculated with porkosan died at one place. At two places in Merseburg, hogs died which, two weeks before, had been inoculated with Pasteur's lymph. At two places in the district of Stade several swine died after being inoculated with Pasteur's lymph. In Kussel 2 swine inoculated with porkosan died of erysipelas. In several communities near Dusseldorf no result was obtained with Pasteur's lymph. In one place the half of 20 inoculated swine are said to have died of erysipelas caused by the lymph. The statistics besides report no herds which showed immunity from disease after preventive inoculation.

**In Tennessee you can scan battlefields that have been the scenes of stirring events in the world's history.**

